# Serotonin/HTR1E signaling blocks chronic stress-promoted progression of ovarian cancer

**DOI:** 10.7150/thno.58956

**Published:** 2021-05-08

**Authors:** Xuan Qin, Jia Li, Shiqing Wang, Jianying Lv, Fangkun Luan, Yanhua Liu, Yanan Chen, Xiaosu Chen, Yujia Zhao, Jingjin Zhu, Yongjun Piao, Wenwen Zhang, Yi Shi, Rong Xiang, Pengpeng Qu, Longlong Wang

**Affiliations:** 1 School of Medicine, Nankai University, Tianjin 300071, China.; 2 Research Institute of Obstetrics and Gynecology, Tianjin Central Hospital of Obstetrics and Gynecology, Tianjin 300100, China.; 3 Department of Gynecological Oncology, Tianjin Central Hospital of Obstetrics and Gynecology, Tianjin 300100, China.

**Keywords:** chronic stress, ovarian cancer, serotonin, HTR1E, SRC

## Abstract

**Rationale:** Psychological stress has been linked to cancer development and resistance to therapy by many epidemiological and clinical studies. Stress-induced immunosuppressive microenvironment by stress hormones, in particular glucocorticoids, has been extensively studied. However, the impacts of other stress-related neurotransmitters, such as serotonin (5-hydroxytryptamine, 5-HT), on cancer development just start to be revealed. Here, we aimed to identify novel neurotransmitters involved in stress-induced growth and dissemination of ovarian cancer (OC) and reveal the major underlying signaling pathway and the therapeutic significance.

**Methods:** Through a genome-wide CRISPR/Cas9 knockout screen in the murine orthotopic model of ovarian carcinoma (OC), we identified candidate genes regulating the peritoneal dissemination of OC. Among them, we picked out HTR1E, one member of 5-HT receptor family specifically expressed in the ovary and endometrium in addition to brain. The correlation of HTR1E expression with OC progression was analyzed in OC patient specimen by quantitative reverse transcription polymerase chain reaction (qRT-PCR), western blot, and immunohistochemistry (IHC). Gain-of-function and loss-of-function analyses were performed to explore the functions of 5-HT/HTR1E signaling in OC growth and dissemination *in vitro* and *in vivo*. In addition, we investigated the therapeutic values of HTR1E specific agonist and small molecular inhibitors against HTR1E downstream factor SRC in a stressed murine OC xenograft model.

**Results:** In OC patients, the HTR1E expression is dramatically decreased in peritoneal disseminated OC cells, which correlates with poor clinical outcome. Silence of HTR1E in OC cells greatly promotes cell proliferation and epithelial mesenchymal transition (EMT) by the activation of SRC-mediated downstream signaling pathways. Furthermore, chronic stress results in significantly decreased serotonin in the ovary and the enhanced OC growth and peritoneal dissemination in mice, which can be strongly inhibited by specific HTR1E agonist or the SRC inhibitor.

**Conclusions:** We discovered the essential role of serotonin/HTR1E signaling in preventing the chronic psychological stress-promoted progression of OC, suggesting the potential therapeutic value of the HTR1E specific agonist and the SRC inhibitor for OC patients who are suffering from psychological stress.

## Introduction

Ovarian cancer (OC) is among the most lethal gynaecological malignancies due to the lack of symptoms at the early stage and hence the occurrence of dissemination at initial diagnosis 1. Psychological stress, including social isolation, anxiety, fear, and depression that most cancer patients may experience during cancer diagnosis and treatment has been shown to promote cancer development and resistance to the therapy by many epidemiological and clinical evidences 2, 3. The high prevalence of depression and anxiety in OC patients accentuates the urgency to understand the molecular mechanisms linking psychological stress to cancer progression 4. Stress-induced hormones, including corticosteroids and catecholamines, through the autonomic nervous system and the hypothalamus-pituitary-adrenal gland axis have been identified as the major neuroendocrine mediators connecting psychological stress to the immune cells, such as T_regs_ 5, myeloid derived suppressor cells 6, dendritic cells 7, 8, and tumor-associated macrophages 9, shaping an immunosuppressive tumor microenvironment that favors cancer development, metastasis, survival, resistance to therapy and recurrence 10. Stress molecules, glucocorticoids in particular, are also able to regulate the metastasis and resistance to chemotherapy of cancer by direct activation of their receptors in cancer cells 11.

In comparison to the extensive studies on glucocorticoid signaling underlying stress-promoted cancer progression, the impacts of other stress-related neurotransmitters, such as serotonin (5-hydroxytryptamine, 5-HT) on cancer development remain largely unexplored. Serotonin is widely distributed in both the central nervous system and peripheral body parts, including the female reproductive tissues, playing multiple roles in the regulation of sleep/wake cycle, mood and emotion, oocyte maturation and being involved in many psychiatric disorders when dysregulated, such as depression and generalized anxiety 12. A nationwide epidemiological study in Denmark reveals that among different classes of antidepressant drugs, only selective serotonin reuptake inhibitors (SSRIs), but not other types of antidepressants that also operate through dopaminergic mechanisms, are associated with a reduced risk of epithelial ovarian cancer 13, the most common type of OC 14, suggesting a potential role of serotoninergic pathways in regulating OC development. Serotonin exhibits both tumor-promoting and tumor-suppressive effects depending on its receptor subtypes in the tumor cells. For example, among 13 serotonin receptor subtypes, HTR2B and HTR1A are implicated in mediating stimulatory effects of serotonin on the initiation and metastasis of prostate cancer, respectively 15, while HTR1B mediates a tumor suppressive role in the initiation of lung cancer, renal cancer, and lymphoma 16.

In a genome-wide CRISPR/Cas9 knock out screen in an orthotopic murine model of OC for essential genes regulating OC progression and peritoneal dissemination, we found one serotonin receptor, i.e., HTR1E, was among the top ten candidate genes. In this study, by combining the mouse stress model with the OC orthotopic mouse model, we reveal the essential effects of HTR1E-mediated downstream signaling of serotonin in suppressing OC growth and peritoneal dissemination, and provide a new mechanism for how stress promotes OC progression.

## Materials and Methods

### Patient samples

20 fresh paired samples (primary samples, ascites samples and metastasis nodules in peritoneal cavity) of OC patients were obtained from Tianjin Center Hospital of Gynecology Obstetrics from December 2017 to December 2019. All the patients were provided with informed consent and met the following conditions: age > 40; high grade serous ovarian adenocarcinoma; TNM stage of Ⅲ-Ⅳ was confirmed by pathologist and clinical doctors. The samples for HE and IHC staining were stored in 4% paraformaldehyde, and the samples for the extraction of protein and RNA were stored in TRIzol reagent. Cancer cells in ascites were harvested by centrifugation and then cultured *in vitro* for the extraction of protein and RNA.

### Cell culture

HEK 293T cell line was obtained from American Type Culture Collection (ATCC, Washington, USA). SK-OV-3, OVCAR-5 and ES-2 cell lines were obtained from the Cell Bank of the Chinese Academy of Sciences (Shanghai, China). Cells were cultured with indicated medium (Biological industries) supplemented with 10% fetal bovine serum (FBS, Gibco), 100 U/mL penicillin and 100 μg/mL streptomycin penicillin in an atmosphere of 5% CO_2_ and 95% air at 37 °C. SK-OV-3 cells were cultured with McCoy's 5A Medium Modified. OVCAR-5 and ES2 cells were cultured with DMEM. HEK 293T cells were cultured with RPMI-1640.

### Establishment of stable cell lines

The coding sequence of HTR1E was synthesized by Sangon Biotech (Shanghai, China) and cloned into plasmid pLV-EF1α-MCS-IRES-Bsd (Biosettia, San Diego, CA, USA). Stable HTR1E overexpressing was conducted in OVCAR-5 cell line. Lentivirus-based RNAi vector pLV-H1-EF1α-puro (Biosettia) with the insertion of shRNA templates were used to conduct HTR1E knocking down in SK-OV-3 cell line. Cells infected with lentivirus for knocking down or reconstituted expression were selected with 4 μg/mL of blasticidin or 10 μg/mL of puromycin (Thermo-Fisher Scientific, Waltham, MA, USA) for one week. The sequences of the shRNA used were as follow: shHTR1E-#1, 5' AAAAGCATGGCTATAAGACCCAAGATTGGATCCA 3'; shHTR1E-#2, 5' AAAAGCCAACTACCTAATCTGTTCTTTGGATCCA 3'; shCtrl, 5' AAAAGCAGTTATCTGGAAGATCAGGTTGGATC 3'.

### Cell counting assay

Cells were seeded into 6-well plates at a density of 5 × 10^4^ cells/well with 2 mL of culture medium containing 10% FBS. The cells were digested and counted every 24 h until they reached nearly 100% confluence. For rescue experiments, cells were incubated at 37 °C with medium containing 10% FBS and serotonin (5 μM, CAS No. 153-98-0, Selleck, Shanghai, China) or SRCi (1 μM, dasatinib, BMS-354825, Selleck, Shanghai, China) or MEKi (1 μM, trametinib, GSK1120212, Selleck, Shanghai, China) at day 2 after cells seeded into 6-well plates.

### Colony formation assay

Cells were seeded into 6-well plates at a density of 1 × 10^3^ cells/well with 2 mL of culture medium containing 10% FBS. For rescue experiments, cells were incubation at 37 °C with medium containing 10% FBS and serotonin (5 μM), SRCi (1 μM) or MEKi (1 μM) at day 8 after cells seeded into 6-well plates. After cultured for two weeks, the colonies on the plates were fixed in 4% paraformaldehyde for 1 h at 4 °C, then stained with crystal violet (C0121, Beyotime) for 8 h, and the numbers of colonies were then counted to evaluate cell proliferation.

### Flow Cytometric Analysis of Cell-Cycle

Cells were seeded the 10 cm dishes at a density of 1×10^6^ cells per dish. When reached nearly 50% confluence, cells were treated with serotonin (5 μM), SRCi (1 μM) or MEKi (1 μM) for 24 h. Subsequently, the cells were harvested, washed with cold PBS, fixed into 70% ethanol at -20 °C for 24 h, stained with 50 mg/mL propidium iodide (PI) (4ABio, China), and analyzed using a Fluorescence-activated cell sorting (FACS) Calibur flow cytometer (BD Biosciences, CA, USA). The results were analyzed using ModFit software (BD Biosciences, CA, USA).

### Cell migration and invasion assays

Cell migration and invasion abilities were measured using Transwell Permeable Supports System (Corning Inc., Corning, NY, USA). A total of 2×10^4^ cells in 200 μL medium containing 2% FBS were seeded into the upper chamber of an 8 μm Millipore transwell inserted in a 24-well plate, and medium containing 10% FBS was added to the bottom chamber. In rescue experiments, cells were incubated for 24 h at 37 °C with medium containing 10% FBS and serotonin (5 μM) or SRCi (1 μM) before seeded in the top Transwell chamber. After 8 h cultured, the bottom Transwell membranes were stained with crystal violet (C0121, Beyotime) for 8 h, and then were washed by PBS and cotton swab. Images were taken using an Olympus BX53 microscope, and the migrated cells were counted in 6 randomly chosen fields to evaluate the migration and invasion activity.

Single cell migration assays were detected by High-Content Imaging and Harmony analysis system (PerkinElmer). 1×10^5^ cells were incubation in a 24-well plate for 24 h at 37 °C with medias containing 10% FBS and serotonin (5 μM) or SRCi (1 μM) before putting into the High-Content Imaging machine.

### Quantitative Real-time PCR

Total RNA from cells and tissues were extracted using TRIzol reagent, and then reverse transcribed into cDNA according to the manufacturer's instructions of M-MLV reverse transcriptase (M1708, Promega). Real-time PCR analyses were performed with LightCycler®96 system (Roche) using SYBR Green SuperMix (Invitrogen) at the recommended thermal cycling settings: 95 °C 300 s, (95 °C 30 s, 60 °C 45 s) × 45 cycles. The relative expression levels of genes were calculated using the 2^-ΔΔCt^ method, and gene expression was normalized to that of β-actin. The primer sequences were listed in Table S1.

### RNA sequencing

Total RNA of HTR1E-kncokdown/HTR1E-overexpressing cells were extracted using TRIzol reagent. The RNA sequencing was performed and analyzed on BGIseq500 platform (BGI-Shenzhen, China). Gene Set Enrichment Analysis (GSEA) was conducted and analyzed based on GSEA software (http://software.broadinstitute.org/gsea/downloads.jsp). The data of differentially mRNA sequencing expressed genes in tumor pathway were transformed using log_2_ transformation.

### Protein extraction and Western blotting

Total protein was extracted from tissues or cells using RIPA lysis buffer with protease inhibitor cocktail and phosphatase inhibitor cocktail (Sigma-Aldrich). Protein was quantified using the Pierce TM BCA Protein Assay Kit (23225, Thermo Scientific) and boiled for 10 min at 95 °C, and then equal amounts of total protein were loaded into Tris-acrylamide gels. Then the proteins were transferred to polyvinylidene fluoride (PVDF) membrane and blocked with 5% defatted milk at room temperature for 1 h. Membranes were incubated with primary antibodies at 4 °C overnight and secondary antibodies (anti-mouse IgG-HRP, ZLI-2305, Zsgb-Bio; anti-rabbit IgG-HRP antibodies, ZLI-2301, Zsgb-Bio) at room temperature for 1 h. The antibodies used in this study are listed in Table S2.

### HE and IHC staining

Tissue specimens of OC patients and mice were fixed in 4% paraformaldehyde, dehydrated in ethanol and then paraffin-embedded. Consecutive sections (6 μm) were performed using microtome, and then deparaffinized in xylene. Tissue sections were stained with hematoxylin (ZLI-9610, Origene) and eosin (ZLI-9613, Origene) (HE) for morphological observations. For IHC staining, 3% H_2_O_2_ was used for antigen retrieval (C1031, Solarbio) and removing peroxidase. 5% goat serum was used for blocking tissue sections before incubated with primary antibodies, biotin-conjugated secondary antibodies (BA-1000, Vector Laboratories), and streptavidin-HRP (SA-5704, Vector Laboratories). The sections were visualized using 3,3'-diaminobenzidine (DAB) substrate (ZLI-9017, Zsgb-Bio), then images were taken using an Olympus BX53 microscope. The degree of immunostaining was measured by H score, and the final score was calculated by multiplying positively stained area (P) with staining intensity (I). The degrees for P were scored as 0-4: 0, < 5%; 1, 5%~25%; 2, 25%~50%; 3, 50%~75%; 4, 75%~100%. The degrees for I were scored as 0 - 3: 0, none; 1, weak; 2, moderate; 3, strong. The antibodies used in IHC staining were listed in Supplementary Table S2.

### Orthotopic murine model

2×10^6^ SK-OV-3 cells or SK-OV-3-shHTR1E/shCtrl cells in 20 μL FBS-Free McCoy's 5A Medium were injected into the right ovary of anaesthetized (5% chloral hydrate) 6-week-old, female NSG mice. Serotonin (5 mg/kg) or BRL54443 (10 mg/kg, CAS No. 57477-39-1, Selleck, Shanghai, China) was into the peritoneal cavity every other day until the mice were sacrificed. Normal saline (20 μL) was used for the injection of the control group. Ascites volumes, the numbers of intestinal metastatic nodules were measured and total cells in ascites were harvested at 6 weeks after OC cells injection. Tumor tissues of mice were collected in TRIzol reagent (Invitrogen) or 4% paraformaldehyde and then store at -80 °C for later experiments. Primary tumor cells and intestinal tumor cells were harvested from NSG mice using PBS and collagenase, then cultured in medium supplemented with 10% fetal bovine serum (Sigma) and 1% Penicillin-Streptomycin (Gibico) at 37 °C in a humidified 5% CO_2_ incubator. Mice were maintained in specific pathogen-free (SPF) facility at room temperature.

### Chronic stress procedures

21 days CUMS model was performed before orthotopic OC murine model. Considering that NSG mice are less tolerant to external stimuli, our chronic stress procedure included: (1) Placed in a humid environment for 24 h every week; (2) Fasting and water deprivation for 24 h every week; (3) Noise stimulus for 2 h every week; (4) Restrained activities for 4 h every day for 3 weeks before tumor transplantation, and continued to be restrained daily for 6 weeks.

### Behavioral tests

The open-field test (OFT) was performed in 2 open boxes (45 cm × 45 cm each) simultaneously, which were separated by opaque closed partition (50 cm high). The behavioral trajectory of mice was recorded by the high-resolution digital camera fixed at center 1 m above the field, and then analyzed through SuperMaze+ detection system. The field area was divided into 9 equal parts, the mice were placed in the center area and then started recording for 5 min. All boxes were wiped with alcohol to remove remaining smell, urine and feces before each new test. All tests were performed within 24 h after 21 days of CUMS model.

The elevated plus maze (EPM) tests were performed in a 4-arm maze with two open arms without walls and two closed arms with walls (25 cm long, 5 cm wide, 60 cm high above the floor). The mouse was placed in the center area and then started recording for 15 min, and then analyzed through Any-maze detection system. A lower frequency of movement and less time spent into center area and open arms indicate higher anxiety. Mice should be acclimatized to the room for 1 h before test, all tests were performed within 24 h after 21 days of CUMS. All areas were wiped with alcohol to remove remaining smell, urine and feces before each new test.

The sucrose preference test (SPT) reflects the dependence of mice on the pleasure of sucrose intake. A decreased intake ratio of sucrose indicates lack of pleasure, which is a core indicator of CUMS model 17. At day 1, 11 and 21 of CUMS model, mice were given access to water and 1% sucrose solution for 2 h at the same time. The preference for sucrose was measured as a percentage of the consumed sucrose solution relative to the total amount of liquid intake.

### ELISA

The blood level of serotonin was measured at the 21^st^ day from the murine caudal vein, 200-500 μL blood was driven from the caudal vein from each mouse with a 1mL syringe. The supernatant was ready to be used for the concentration assay of serotonin after the blood was centrifuged. The serotonin concentration of murine was assayed by murine serotonin ELISA Kit (EIA-2440, Shanghai Youxuan Sci & Tech Development) as the protocols described. The OD values were obtained from microplate reader (Infinite M200 PRO, TECAN) at 450 nm. The data were analyzed by Curve Expert, and adjusted for the protein concentrations.

### Nissl and Golgi staining

Mice of stress and control groups were perfused through the heart with 4% paraformaldehyde after deeply anesthetized. For Nissl staining, brain tissues were fixed overnight in 4% paraformaldehyde, dehydrated in ethanol and then paraffin-embedded. Consecutive sections (15 μm) were performed using microtome, and then stained in 0.1% toluidine blue solution for 20 min, washed by Milli-Q water and dehydrated in ethanol. For Golgi staining, brain tissues were sliced into coronal sections (200 μm) and then stained following the manufacturer's instructions of FD Rapid GolgiStain^TM^ Kit (FD Neuro Technologies, Inc., USA). The images of Nissl and Golgi staining were captured using an Olympus BX53 microscope.

### Pharmacologic studies

After 3 days of tumor transplantation on NSG mice, studies of drug treatment through intraperitoneal injection were performed. The powder of serotonin (Selleck, 5 mg/kg, qod) and BRL54443 (Selleck, 10 mg/kg, qod) were dissolved in 0.9% sterile saline and stored at 4 °C. The treatment lasted for 6 weeks until the mice were sacrificed.

### Statistics

Graphpad Prism 8.0 software was used for statistical analysis. Quantitative data were presented as mean ± SEM and statistical analysis was performed as described in the legends. Survival curves were estimated using the Kaplan-Meier method, and the log-rank test was used to calculate differences between the curves.

### Ethics approval and consent to participate

The Helsinki Declaration was strictly followed regarding the use of OC patients' samples. The patients study received ethical approval from the ethics committee of Nankai University and Tianjin Center Hospital of Gynecology Obstetrics. All animal experiments were approved by Institutional Animal Care and Use Committee of Nankai University.

## Results

### Genome-wide CRISPR screen for potential suppressors of OC identifies that HTR1E is dramatically decreased in the peritoneal disseminated OC cells

**Figure 1:**
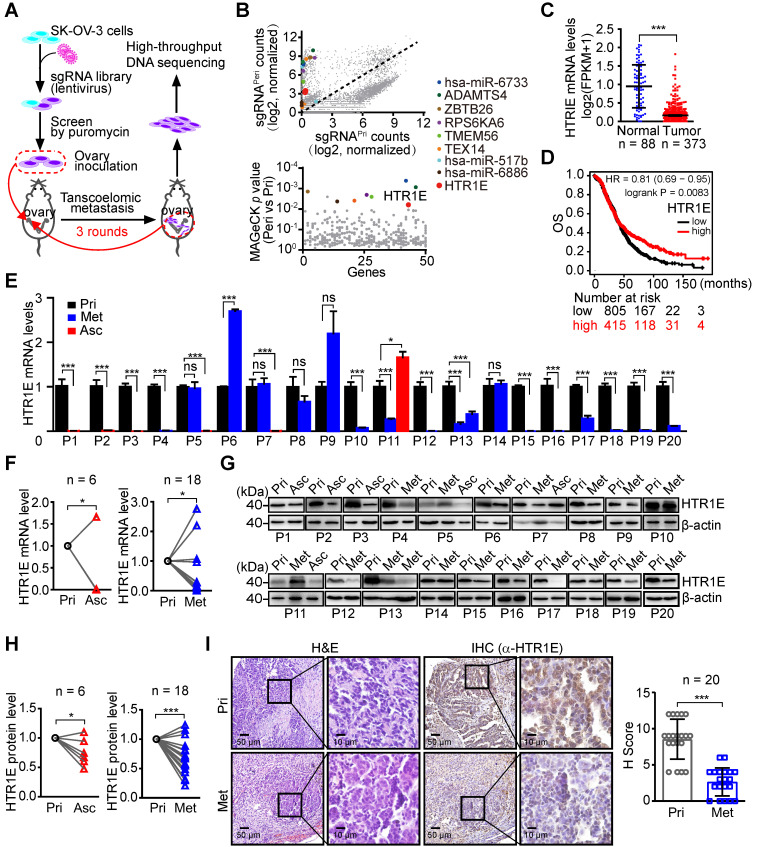
** Genome-wide CRISPR screen identifies HTR1E as a key gene regulating OC peritoneal dissemination. (A)** The schematic diagram of genome-wide *in vivo* screening of key genes regulating peritoneal dissemination by CRISPR/Cas9 library in an OC orthotopic murine model. **(B)** Scatterplot showing enrichment of specific sgRNAs in peritoneal disseminated (sgRNA^Peri^) or primary (sgRNA^Pri^) OC xenografts (top panel) and the identification of top candidate genes using MAGeCK P value analysis (bottom panel). **(C)** The mRNA level of HTR1E in normal human ovarian tissues (n = 88) and OC specimens (n = 373) from Genotype-Tissue Expression (GTEx) database and TCGA database (***P < 0.001, by paired, two-tailed student's t-test). **(D)** Kaplan-Meier survival curve to show the overall survival (OS) of OC patients with different HTR1E expression (n = 1220). **(E-F)** qRT-PCR analysis of HTR1E in 20 human OC specimens (P) including 20 primary tumor samples (Pri) with 6 paired ascites samples (Asc) and 17 paired peritoneal OC metastatic nodules (Met) **(E)** and the statistical analysis on the change of HTR1E expression during OC metastasis **(F)** (means ± SEM from three independent experiments, *P < 0.05, ns not significant, by paired, two-tailed student's t-test). **(G-H)** Western blot analysis of HTR1E in human OC primary tumors and paired metastases and ascites from 20 OC patients **(G)** and the statistical analysis on the change of HTR1E protein during OC metastasis **(H)** (*P < 0.05, ***P < 0.001, by paired, two-tailed student's t-test). **(I)** H&E and IHC staining of HTR1E in the primary OC tumors and paired metastases dissected from human OC patients and the quantification by H score method (n = 20, ***P < 0.001, by paired, two-tailed student's t-test).

To systematically identify essential genes that regulate the transcoelomic metastasis of OC, we used GeCKO (v2) sgRNA pooled libraries to perform genome-wide gene knockout in SK-OV-3 cells 18. SK-OV-3 cells are well differentiated serous adenocarcinoma cells and can form xenograft tumors with a histology that is also very similar to a human serous ovarian cancer 19, 20. We screened cancer cells with highly peritoneal metastatic capacity in an orthotopic murine OC model and performed a high throughput DNA sequencing analysis (Figure 1A). Among the top ten sets of sgRNAs enriched in peritoneal disseminated OC cells, which are analyzed by Model-based Analysis of Genome-wide CRISPR/Cas9 Knockout (MAGeCK) algorithm 21, we find two sgRNAs targeting a receptor of the stress-related neurotransmitter serotonin, i.e., HTR1E (Figure 1B, Table S3), which is abundantly expressed in the ovary and stomach in addition to the brain (Figure S1A). Furthermore, RNA profiling results in the GTEx database and TCGA database show that among the serotonin receptor family, HTR1E is the most abundant subtype expressed in normal human ovary, which shows dramatic decrease in OC (Figure S1B, Figure 1C). HTR1E also shows dramatic decrease in glioblastoma and stomach adenocarcinoma, while HTR1E is highly expressed in the normal brain and stomach (Figures S1A and S1C). And in OC patients, higher expression of HTR1E correlates with better clinical outcome (Figure 1D). These results suggest that HTR1E is a potential tumor suppressor in ovary, brain and stomach.

To further investigate the differential HTR1E expression during the peritoneal dissemination of OC, we performed quantitative RT-PCR (qRT-PCR) analyses of HTR1E in 20 OC patients who had peritoneal dissemination and some of them had malignant ascites formed. The results show dramatically decreased HTR1E expressions in disseminated OC cells in both malignant ascites and peritoneal metastatic lesions (Figures 1E-1F). Consistently, significantly decreased expressions of HTR1E protein were revealed by western blot (Figures 1G-1H) and immunohistochemistry (IHC) analyses (Figure 1I). Interestingly, among serotonin receptor family, only HTR1E significant decreases during peritoneal dissemination (Figure S1D).

HTR1E also shows lower expression in a high-grade serous OC cell line OVCAR-5 22, 23 than that in low metastatic SK-OV-3 among the major serotonin receptors (Figure S1E). In an orthotopic murine model of OC established by the inoculation of SK-OV-3, a few xenograft-bearing mice have very limited peritoneal metastatic lesions, where HTR1E shows dramatic decrease when compared with that in the primary tumor sites among the major serotonin receptors (Figure S1F), suggesting a potential role of HTR1E in preventing the peritoneal dissemination of OC.

### HTR1E plays a suppressive role on the growth and dissemination of OC in mice

**Figure 2:**
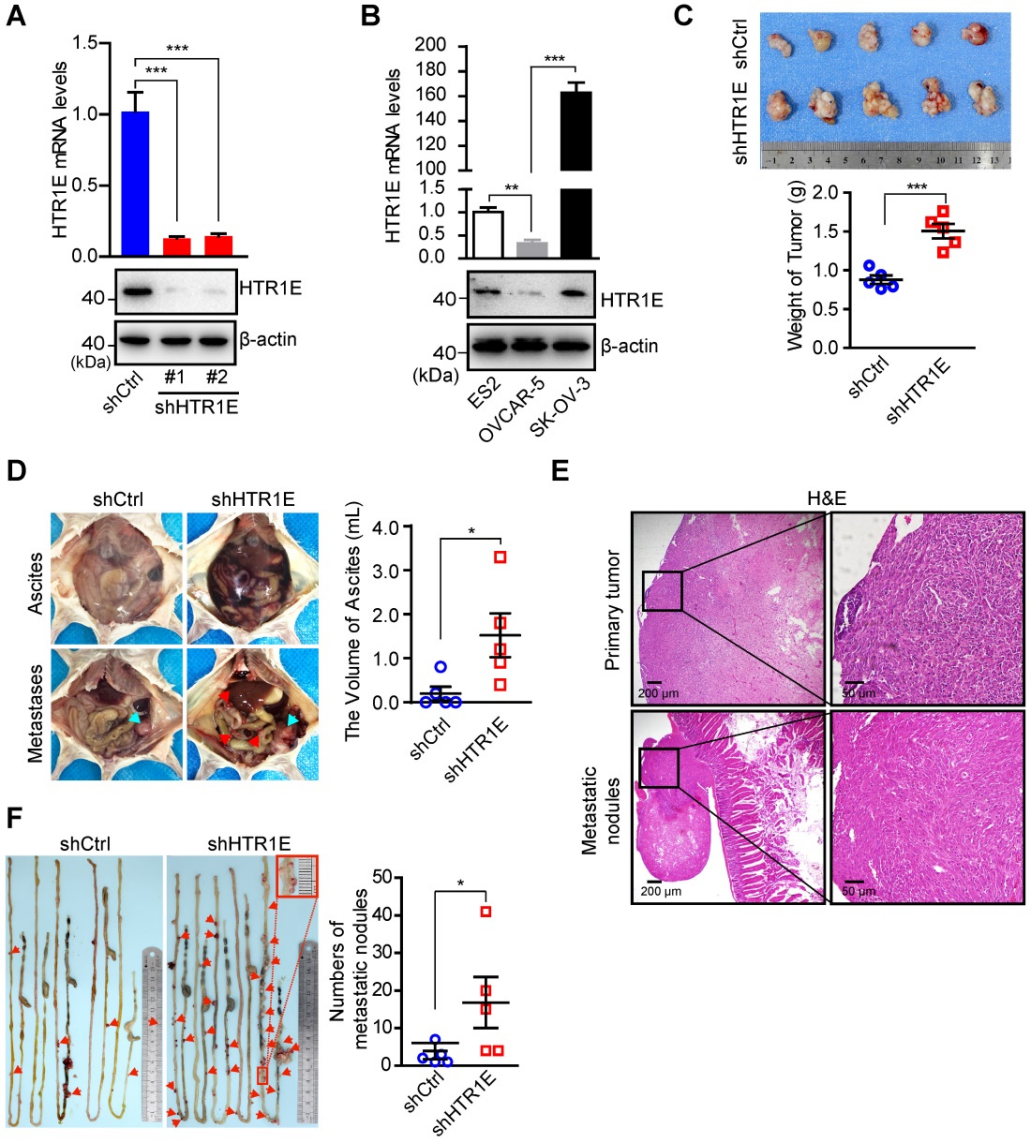
** HTR1E inhibits the growth and peritoneal dissemination of OC xenografts in mice. (A)** qRT-PCR and western blot showing the knockdown efficiencies of shRNAs targeting HTR1E (shHTR1E) (means ± SEM from three independent experiments, ***P < 0.001, by unpaired, two-tailed student's t-test). The shRNA targeting bacteria lacZ is used as a negative control (shCtrl). **(B)** qRT-PCR and western blot analysis of HTR1E in indicated OC cell lines (means ± SEM from three independent experiments, **P < 0.01, ***P < 0.001, by unpaired, two-tailed student's t-test). **(C)** Images of OC xenografts dissected from NSG mice orthotopically inoculated with indicated SK-OV-3 cells 42 days ago and the weights of the xenografts (n = 5 for each group, ***P < 0.001, by unpaired, two-tailed student's t-test). **(D)** Representative images of the abdominal parts of mice (left). The primary tumors and the metastatic nodules are indicated by blue and red arrows, respectively. The volumes of ascites in the peritoneal cavities were measured (right, n = 5 for each group, *P < 0.05, by unpaired, two-tailed student's t-test). **(E)** H&E staining of the primary OC xenografts and the metastatic nodules on the intestine. (F) Representative images of peritoneal metastatic nodules on the intestines (left) and the quantification (right, n = 5 for each group, *P < 0.05, by unpaired, two-tailed student's t-test).

To investigate the biological significance of HTR1E in OC progression, we knock down HTR1E in SK-OV-3 cells (Figure 2A), which have relatively high level of HTR1E when compared with OVCAR-5 cells (Figure 2B), and inoculated these cells orthotopically into the immunodeficient NOD/SCID (NSG) mice. Knocking down HTR1E results in faster growth of tumor xenografts (Figure 2C). In addition, mice bearing HTR1E-silenced xenografts have much more malignant ascites formed in the peritoneal cavity (Figure 2D), which is accompanied by significantly increased numbers of peritoneal disseminated tumor nodules mainly found on the surfaces of intestines in the peritoneal cavity (Figures 2E-2F).

**Figure 3:**
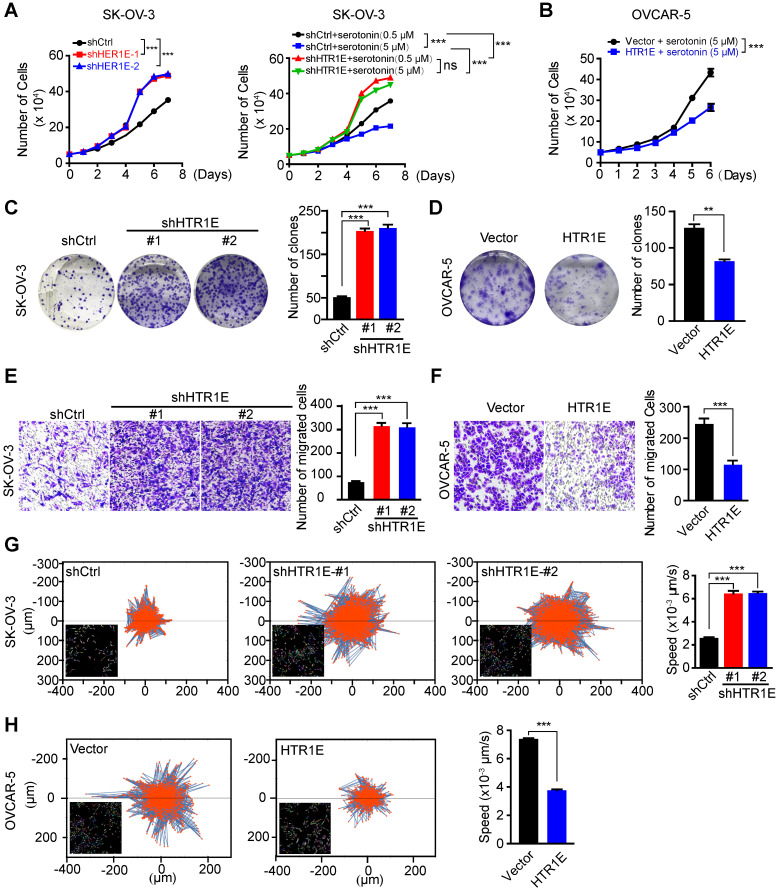
** HTR1E inhibits the proliferation and migration of OC cells. (A)** The proliferation curves of SK-OV-3 cells transfected with indicated shRNAs in the medium containing basal level of serotonin (0.5 µM) (left) or 5 µM serotonin (right, means ± SEM from three independent experiments, ***P < 0.001, by two-way ANOVA test). **(B)** Cell proliferation curves of OVCAR5 cells transfected with empty vector (vector) or HTR1E and cultured in the presence of 5 µM serotonin (means ± SEM from three independent experiments; ***P < 0.001, by two-way ANOVA test). **(C-D)** Colony formation assay of SK-OV-3** (C)** and OVCAR-5 **(D)** cells in the presence of 5 µM serotonin and the quantification. Data are shown as means ± SEM from three independent experiments (**P < 0.01, ***P < 0.001, by unpaired, two-tailed student's t-test). **(E-F)** Transwell cell migration assay of SK-OV-3 **(E)** and OVCAR-5 **(F)** cells in the presence of 5 µM serotonin and the quantification. Data are shown as means ± SEM from three independent experiments (***P < 0.001, by unpaired, two-tailed student's t-test). **(G-H)** Trajectory of single SK-OV-3 **(G)** and OVCAR-5 **(H)** cell cultured in the presence of 5 μM serotonin. The tracked cells of current displacement and average speeds of all cells are shown as means ± SEM from three independent experiments (***P < 0.001, by unpaired, two-tailed student's t-test).

In SK-OV-3 cells cultured in the complete medium containing basal level of serotonin (0.5 μM) from fetal bovine serum (FBS), knocking down HTR1E significantly promotes the proliferation of SK-OV-3 cells (Figure 3A, left panel). The addition of more serotonin (5 μM) is able to further inhibit the proliferation of control SK-OV-3 cells, while in HTR1E-silenced cells, the additional serotonin-induced inhibition on cell proliferation is dramatically attenuated (Figure 3A, right panel). In addition, reconstitution of HTR1E in OVCAR-5 cells (Figure S2A), that have much lower level of endogenous HTR1E, significantly inhibits their proliferation in the medium containing basal level of serotonin (0.5 μM) (Figure 3B). The results of the colony formation assay confirm that inhibition of HTR1E strongly enhances colony formation capacity of SK-OV-3 cells (Figure 3C), while reconstitution of HTR1E reduces colony formation of OVCAR-5 cells (Figure 3D). Furthermore, reconstitution of HTR1E in OVCAR-5 induces the cell cycle arrest in G1 phase and decreases the numbers of cells in S and M phases (Figure S2B). These data suggest that HTR1E mediates the major inhibitory effect of serotonin on cell proliferation.

Moreover, knocking down HTR1E significantly increases the motility of SK-OV-3 cells shown by the transwell migration assay (Figure 3E), while reconstitution of HTR1E in OVCAR-5 cells decreases their motilities (Figure 3F). The results of single cell migration monitered by the high-content imaging and analysis system suggest increased current displacement and average speeds in HTR1E-silenced SK-OV-3 cells (Figure 3G), and decreased current displacement and average speeds in HTR1E-reconstituted OVCAR-5 cells (Figure 3H).

Taken together, these data suggest that HTR1E is the major receptor mediating a suppressive role of serotonin on the growth and peritoneal dissemination of OC by inhibiting the proliferation and migration of OC cells.

### Serotonin/HTR1E negatively couples to SRC to inhibit cell proliferation and epithelial mesenchymal transition (EMT)

**Figure 4:**
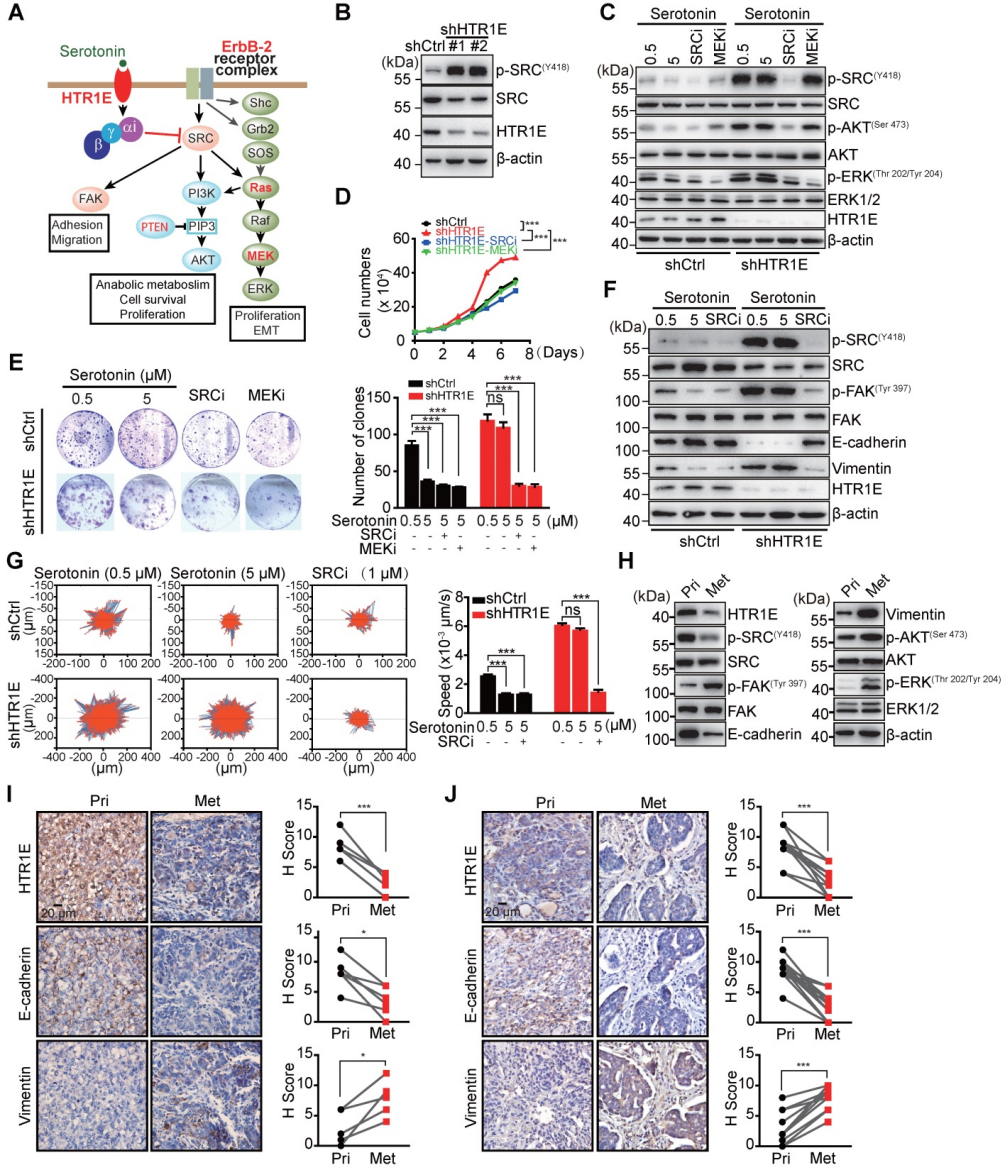
** HTR1E inhibits SRC-mediated pathways that promote cell proliferation and EMT. (A)** Hypothesized signaling pathways triggered by serotonin/HTR1E based on GSEA analysis. **(B)** Western blot analysis of the activation of SRC in shHTR1E or shCtrl SK-OV-3 cells. **(C)** Western blot analysis of the activation of SRC and ERK in SK-OV-3 cells in the presence of serotonin and SRC inhibitor (SRCi, 1 µM) or MEK inhibitor (MEKi, 1 µM). **(D)** Cell proliferation curves of SK-OV-3 cells in the presence of serotonin (5 µM) with or without SCRi (1 µM) or MEKi (1 µM) (means ± SEM from three independent experiments, ***P < 0.001, by two-way ANOVA test). **(E)** Colony formation assays of SK-OV-3 cells in the presence of serotonin with or without SCRi (1 µM) or MEKi (1 µM) (means ± SEM from three independent experiments, ***P < 0.001, ns not significant, by unpaired, two-tailed student's t-test). **(F)** Western blot analysis of EMT markers in indicated SK-OV-3 cells in the presence of serotonin and SRCi (1 µM). **(G)** The motility of indicated SK-OV-3 cells in the presence of serotonin and SRCi (1 µM) analyzed by High-Content Imaging and Harmony analysis system (means ± SEM from three independent experiments, ***P < 0.001, ns not significant, by unpaired, two-tailed student's t-test). **(H-I)** Western blot **(H)** and IHC **(I)** analysis of the EMT markers and the activation of SRC and ERK in the primary OC xenografts (Pri) and the peritoneal metastases (Met) dissected from the SK-OV-3 orthotopic murine model of OC. The quantification by H score method is analyzed by paired, two-tailed student's t-test (n = 6, *P < 0.05, ***P < 0.001). **(J)** IHC analysis of human primary OC specimens (Pri) and paired peritoneal metastases (Met) for HTR1E and EMT markers. The correlation between HTR1E and EMT markers is analyzed by paired, two-tailed student's t-test (n = 13, ***P < 0.001).

To get insights into the HTR1E-mediated downstream signaling in OC cells, we performed transcriptome analysis on HTR1E-silenced SK-OV-3 cells. Gene sets enrichment analysis (GSEA) shows that silencing HTR1E mainly upregulates hallmark genes in EMT and several metabolic pathways, including glycolysis and lipid metabolism (Figure S3A). EMT has been demonstrated to play essential roles in the peritoneal dissemination of OC cells 24-26. Knocking down HTR1E leads to the upregulation of many genes responsible for extracellular matrix organization and EMT-driving transcriptional factors (Figures S3B-S3C), contributing to the enhanced motility of HTR1E-silenced SK-OV-3 cells. Further GSEA analysis on oncogenic signatures reveals that silencing HTR1E upregulates gene sets that are also positively regulated by ERBB2 (also known as HER2) downstream signals, including SRC-RAS-RAF-MEK-ERK and SRC-PI3K-AKT, which is negatively regulated by PTEN (Figures S3D-S3F). Given that SRC is a direct effector of G proteins and HTR1E belongs to the G-protein-coupled receptors (GPCRs) superfamily that negatively couples to the adenylyl cyclase (AC) through G proteins for specific signal transduction 27, 28, we postulates that serotonin/HTR1E may signal through SRC to regulate downstream RAS-RAF-MEK-ERK and PI3K-AKT pathways to eventually affect cell proliferation and EMT (Figure 4A).

When cultured in the medium containing basal level of FBS-derived serotonin (0.5 μM), HTR1E-silenced SK-OV-3 cells have dramatically activated SRC shown by its phosphorylation (Figure 4B**)**. In control SK-OV-3 cells with relatively high level of HTR1E, serotonin inhibits SRC phosphorylation in a dose-dependent manner, while in HTR1E-silenced cells, serotonin is no longer able to inhibit SRC activation (Figure 4C). Knocking down HTR1E also results in the activation of ERK, which can be inhibited by either the SRC inhibitor or the MEK inhibitor (Figure 4C), suggesting that serotonin signals through HTR1E to inhibit the activation of SRC and downstream MEK-ERK pathway. Inhibition of either SRC or MEK by the specific inhibitors significantly decreases the proliferation and colony formation capacity of HTR1E-silenced SK-OV-3 to the levels comparable with that of the control cells in the presence of serotonin (Figures 4D-4E). In addition, cell-cycle analysis reveals that inhibition of either SRC or MEK can dramatically arrest HTR1E-silenced SK-OV-3 cells in G1 phase (Figures S4A-S4B). In OVCAR-5 cells that with much less endogenous HTR1E, reconstitution of HTR1E by ectopically expressed proteins dramatically inhibits the activation of SRC and ERK (Figure S5A), which consequently inhibits the proliferation of OVCAR-5 cells to the rate comparable to that of specific SRC and ERK inhibitors (Figure S5B). These data suggest that serotonin/HTR1E signaling regulates cell proliferation mainly through SRC-MEK-ERK pathway.

In control SK-OV-3 cells, serotonin is able to dose-dependently increase the level of epithelial marker E-cadherin and decreases the mesenchymal marker vimentin, which indicates an inhibited EMT process, while in HTR1E-silenced cells serotonin can no longer inhibits the EMT (Figure 4F), suggesting that serotonin inhibits the EMT process through HTR1E-mediated signaling. Consistently, serotonin inhibits the mobility of control SK-OV-3 cells in a dose-dependent manner, while in HTR1E-silenced cells serotonin is not able to affect the cell mobility (Figure 4G). And the enhanced EMT and cell mobility by silencing HTR1E can be completely blocked by the specific SRC inhibitor (Figures 4F-4G). In HTR1E-deficient OVCAR-5 cells, reconstitution of HTR1E also inhibits EMT process shown by dramatically increased E-cadherin expression and decreased vimentin (Figure S5C).

In the orthotopic murine model of OC, the decrease of HTR1E in the peritoneal metastatic OC cells is concurrent with the enhanced activation of SRC-ERK pathway and EMT when compared with that in OC cells at the primary sites (Figures 4H-4I), which is also observed in human OC specimen (Figure 4J). Taken together, these data suggest that serotonin/HTR1E signals through SRC in OC cells to inhibit cell proliferation and EMT.

### Serotonin inhibits the growth and peritoneal dissemination of OC in mice through HTR1E-mediated signaling

**Figure 5:**
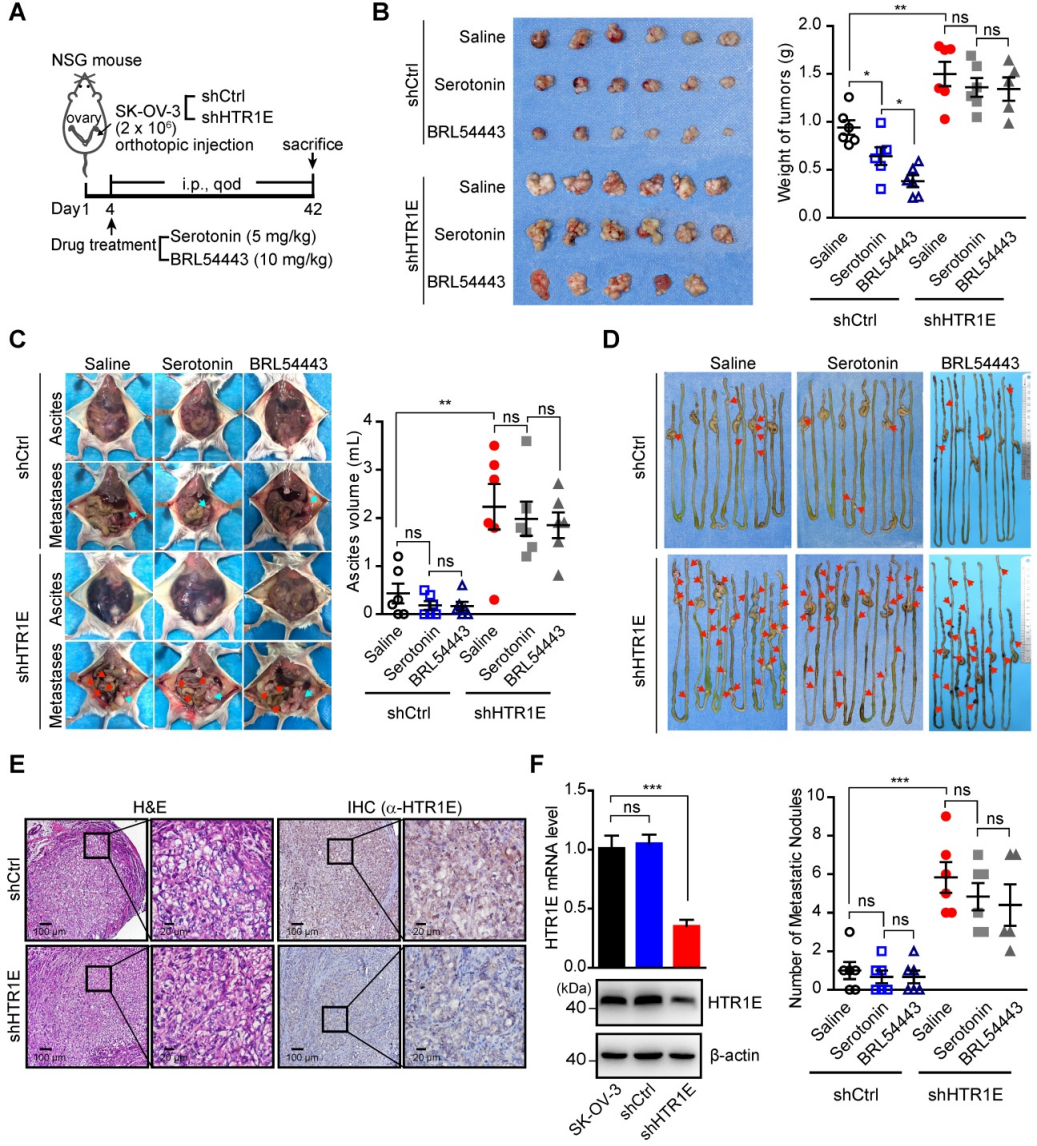
** Serotonin inhibits OC development in mice in an HTR1E-dependent manner. (A)** Schematic of the experiment. **(B)** Images of the primary OC xenografts formed by orthotopic inoculation of HTR1E-silenced or control SK-OV-3 cells with indicated treatments (left) and the tumor weights (right) (shown as means ± SEM; n = 6 for each group; *P < 0.05, **P < 0.01, ns not significant, by unpaired, two-tailed student's t-test). **(C)** Representative images of the abdominal parts of mice with indicated OC xenografts (left) and the quantification of the ascites volumes (right; shown as means ± SEM; n = 6 for each group; **P < 0.01, ns not significant, by unpaired, two-tailed student's t-test). **(D)** Images of OC metastatic nodules on the intestines (top panel) and the quantification (bottom panel; shown as means ± SEM; n = 6 for each group; ***P < 0.001, ns not significant, by unpaired, two-tailed student's t-test). **(E)** H&E and IHC staining of HTR1E in the primary OC xenografts formed by orthotopic inoculation of HTR1E-silenced or control SK-OV-3 cells. **(F)** qRT-PCR and western blot showing the knock-down efficiencies of shRNAs targeting HTR1E (shHTR1E) in SK-OV-3 cells used in the orthotopic murine model of OC (means ± SEM from three independent experiments, ***P < 0.001, by unpaired, ns not significant, two-tailed student's t-test).

Given the essential roles of serotonin in the ovary for oocyte maturation, the abundant expression of HTR1E in the normal ovary, and the dramatic decreased HTR1E in OC progression, we postulated that serotonin might have a tumor suppressive role mediated by HTR1E to prevent the malignant development of OC. To test, we treated the mice bearing orthotopically transplanted SK-OV-3 xenografts with serotonin (Figure 5A). As Figure 5B shows, injection of serotonin and HTR1E specific agonist BRL54443 significantly inhibit the growth of OC xenografts in the control group, while has no effect on HTR1E-silenced OC xenografts. In addition, serotonin and BRL54443 also slightly reduce the formation of malignant ascites and peritoneal dissemination, although not statistically significantly, that is possibly due to that the high level of local serotonin produced by the enterochromaffin cells in the gastrointestinal tract that saturates HTR1E (Figures 5C-5D). Also, serotonin and BRL54443 treatment show no further inhibitory effect on the ascites formation (Figures 5C-5D) and the peritoneal dissemination of OC cells, whose HTR1E proteins are stably knocked down (Figures 5E-5F). These data suggest that serotonin can suppress the growth and dissemination of OC via HTR1E-mediated signaling. And the blockage of HTR1E-mediated downstream signaling of serotonin by decreasing the expression of HTR1E might be a prerequisite for the growth of OC. This blockage of serotonin/HTR1E signaling is possibly more essential for the formation of peritoneal OC metastatic colonies on the surface of gastrointestinal tract where high level of serotonin is produced. The dramatically reduced HTR1E in metastatic OC lesions compared with that in the OC cells from primary sites supports this hypothesis.

### Chronic stress promotes OC progression by interfering with serotonin/HTR1E signaling

**Figure 6:**
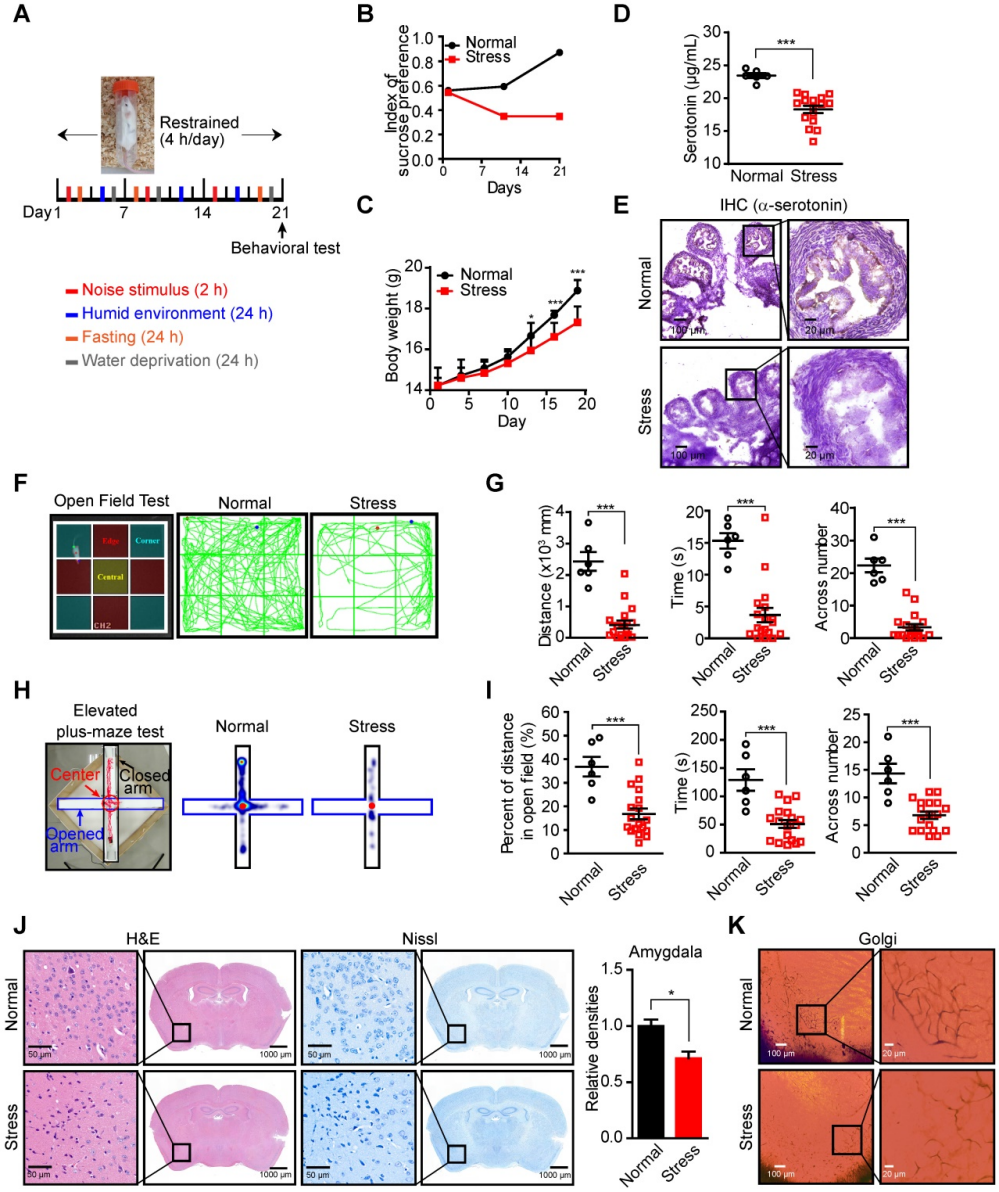
** Decreased serotonin in the peripheral blood serum and ovary of mice in CUMS model. (A)** Schematic of the CUMS model. (B-C) Sucrose consumption ratio in the sucrose preference test **(B)** and body weights **(C)** (n = 6 in Normal group, n = 18 in Stress group, shown as means ± SEM; *P < 0.05, ***P < 0.001, by unpaired, two-tailed student's t-test). **(D)** Serum serotonin levels measured by ELISA (n = 6 in Normal group, n = 18 in Stress group, shown as means ± SEM; ***P < 0.001, by unpaired, two-tailed student's t-test). **(E)** Ovary serotonin levels measured by IHC staining in mice with or without stress stimulation for 21 days. **(F)** Zoning diagram of open-field test (left) and representative locomotion tracks (green lines) of mice in the normal group and mice under stress (right). **(G)** The locomotion distance, residence time and the number across center area are compared between mice in the normal group and stress group (shown as means ± SEM; ***P < 0.001, by unpaired, two-tailed student's t-test). **(H)** Zoning diagram of elevated high-plus maze test (left) and representative locomotion heat map of mice in normal group and stress group (right). **(I)** The time spent in the open arm area and the number across the center area are compared (shown as means ± SEM; ***P < 0.001, by unpaired, two-tailed student's t-test). **(J)** H&E and Nissl staining of neuron cells in amygdala of mice in normal and stress groups (left). The quantification of relative densities of neuron cells in amygdala of mice (shown as means ± SEM; *P < 0.05, by unpaired, two-tailed student's t-test). (K) Golgi staining of nerve synapse in amygdala of mice in normal and stress groups.

As an important neurotransmitter involved in many psychiatric disorders, such as depression and anxiety, the levels of neural and serum serotonin are affected by the psychologic stress 29, 30, which may lead to the fluctuation of local serotonin levels in the ovary since the majority of ovarian serotonin originates from the uptake of serum serotonin 31, 32. Decreased serum serotonin has been detected in a murine stress model 8, 33, 34, suggesting that stress may reduce local serotonin in the ovary and affect serotonin/HTR1E-mediated tumor suppressive effects. To test this hypothesis, we applied the chronic unpredictable mild stress (CUMS), a widely used animal model of depression 35-37. We performed daily irregular stress stimulation on mice for 3 weeks before the orthotopic inoculation of SK-OV-3 cells and the restrained stimulation (4 h/day) continued thereafter (Figure 6A). The mice showed anxiety-like behavior after 3-week stress stimulation, which was confirmed by the decreased index of sucrose preference (Figure 6B) and the loss of body weights (Figure 6C). And consistent to the reported results 8, 34, chronic stress decreases the serotonin level in serum and ovary of mice (Figure 6D). The local serotonin level of mouse ovary also decreased possibly affected by peripheral blood (Figure 6E), as the transportation and synthesis of serotonin in mouse ovary have no difference between these two groups (Figures S6A-S6B). In behavioral tests, the mice of the stress group also showed less open field exploration behavior in the open-field test (Figures 6F-6G) and the decreased number of entries into the open arms, the time and the distance spent there in the elevated high-plus maze test (Figures 6H-6I). Analysis of the pathologic changes in the left amygdala, which plays a critical role in generating fear and persistent anxiety 38, 39, by H&E and Nissl staining for decreased neuron cells and Golgi staining for sparse nerve synapse also confirm the successful stress status established in the mice (Figures 6J-6K).

**Figure 7:**
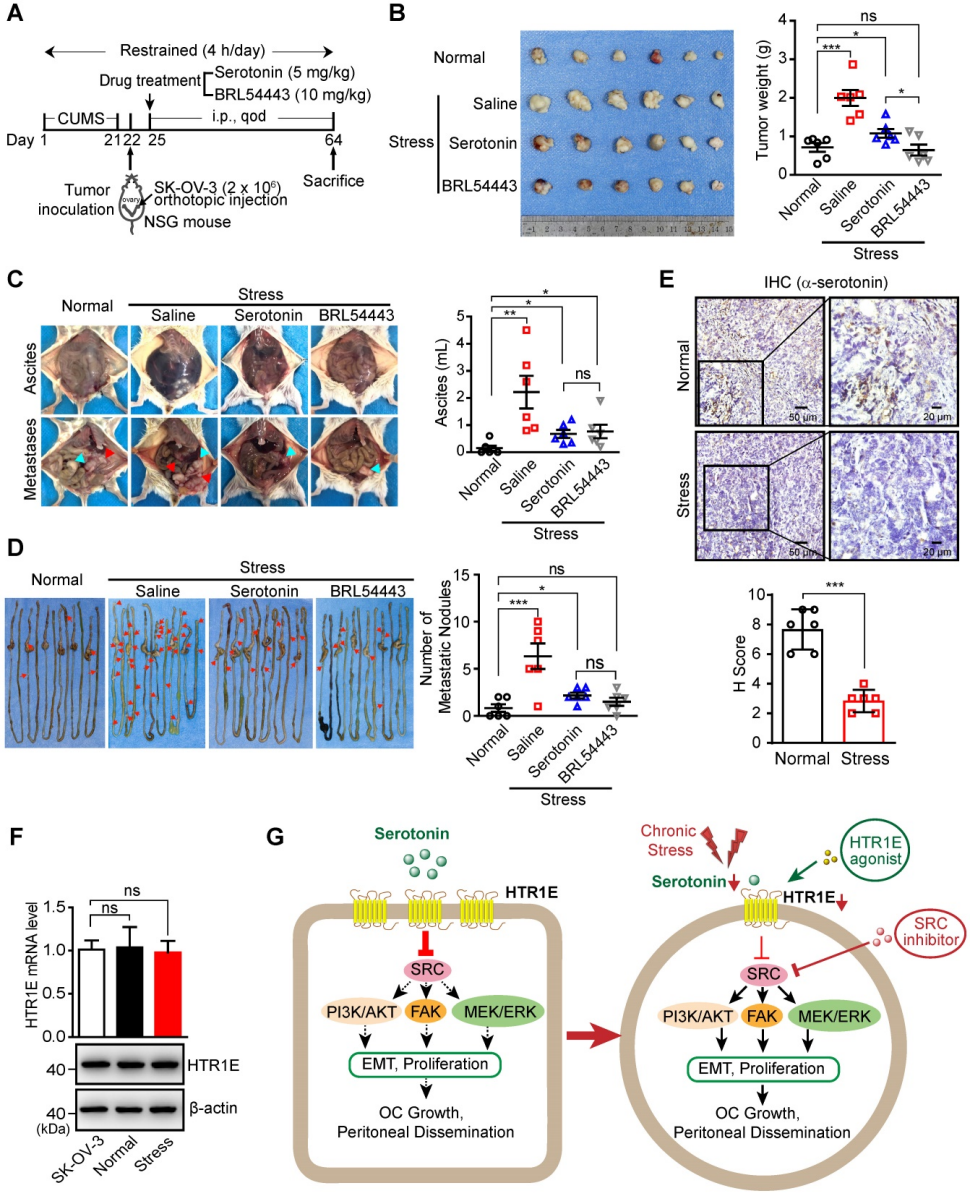
** Activation of Serotonin/HTR1E signaling reduces chronic stress-induced OC progression in mice. (A)** Schematic of the experiments (n = 6 in Normal group, n = 18 in Stress group). **(B)** Images of OC xenografts at the primary sites dissected from SK-OV-3 orthotopic murine model of OC with indicated treatments (left) and the tumor weights (right; n = 6 in each group; shown as means ± SEM; P < 0.05, ***P < 0.001, ns not significant, by unpaired, two-tailed student's t-test). **(C)** Representative images of the abdominal parts of mice with OC xenografts under indicated treatments (left) and the quantification of the ascites volumes (right; n = 6 in each group; shown as means ± SEM; *P < 0.05, **P < 0.01, ns not significant, by unpaired, two-tailed student's t-test). **(D)** Images of the metastatic nodules on the intestines of mice with OC xenografts under indicated treatments (left) and the quantification (right; n = 6 in each group; shown as means ± SEM; *P < 0.05, ***P < 0.001, ns not significant, by unpaired, two-tailed student's t-test). **(E)** Serum serotonin levels measured by IHC staining (top panel) in OC mice with or without stress stimulation and the quantification (bottom panel) by H score method (n = 6, ***P < 0.001, by unpaired, two-tailed student's t-test). **(F)** qRT-PCR and western blot showing the HTR1E mRNA and protein levels of tumor tissues in the orthotopic murine model of OC (means ± SEM from three independent experiments, ns not significant, two-tailed student's t-test). **(G)** Schematics summarizing the HTR1E-mediated signaling of serotonin in preventing the progression of OC.

In the orthotopic murine model of OC (Figure 7A), chronic stress significantly promotes the growth of OC xenografts (Figure 7B), the formation of malignant ascites (Figure 7C) and peritoneal metastatic nodules on the surface of gastrointestinal tract (Figure 7D), which are able to be blocked by the injection of serotonin to restore serotonin level under stress (Figures 7B-7D). Stress-promoted OC growth and peritoneal dissemination can also be inhibited by the specific agonist of HTR1E, i.e., BRL54443 40, 41, strongly suggesting that HTR1E-mediated downstream pathway of serotonin plays the major suppressive role against stress-induced tumor growth and dissemination (Figures 7B-7D). Interestingly, BRL54443 exhibits stronger activity than serotonin to suppress the growth of OC xenografts at the primary sites (Figure 7B). This is possibly due to the tumor-promoting roles of other serotonin receptors, such as HTR1D 42 and HTR7 43, that are slightly upregulated in OC (Figures S1B and S1D) and can activate the SRC signaling. However, BRL54443 does not show further suppressive activity than serotonin against the peritoneal dissemination of OC and ascites formation (Figures 7C-7D), possibly due to the lower expression of HTR1E in metastasized SK-OV-3 cells and relatively high level of serotonin in gastrointestinal tract that saturates HTR1E. There is no toxicity of serotonin and BRL54443 treatment to major organs when evaluated by H&E staining of heart, liver, spleen, lung, kidney and brain (Figure S7). We also observed decreased serotonin level in the xenograft tumor of mice under chronic stress (Figure 7E), while there was no change on HTR1E expression under chronic stress (Figure 7F).

Taken together, these results suggest that chronic stress-induced decrease of local serotonin in the ovary and hence the attenuation of downstream HTR1E-mediated tumor suppressive signaling may result in increased OC growth and dissemination. And reactivation of serotonin/HTR1E to inhibit downstream SRC-mediated tumor promoting signaling pathways by the specific HTR1E agonist or the specific SRC inhibitor is a promising strategy to inhibit chronic stress-promoted OC growth and dissemination (Figure 7G).

## Discussion

Chronic psychological stress has long been recognized as a risk factor to stimulate the development of many types of cancer including OC, behind which stress hormones, particularly catecholamines and corticosterones, are revealed to play major roles by promoting an immune-suppressive microenvironment that supports the cancer cell survival, metastasis and drug resistance. Here, we reveal a novel direct mechanism linking the chronic stress through the serotonergic neurotransmitter to OC cells by HTR1E, an ovary-specific serotonin receptor subtype outside the brain. We found that in OC cells, HTR1E-mediated downstream signaling of serotonin strongly inhibits the activation of SRC, which is a central mediator of many oncogenic signaling from tyrosine kinase receptors such as ERBB2 (also known as HER2), eventually resulting in the dramatically attenuated MEK-ERK, PI3K-AKT, and FAK pathways and inhibited cell proliferation and EMT process, a key mechanism for the peritoneal dissemination of OC cells. Besides its canonical roles as a neurotransmitter in the nervous system, serotonin is also a key factor for egg maturation in mammalian ovaries 12. Given the existence of several subtypes of serotonin receptors in the ovarian cells, such as HTR2A, HTR2B, and HTR7 (Figure S1B), that may couple to PI3K-AKT, RAS-MEK-ERK signaling pathways to promote cell survivor, proliferation and migration 44, HTR1E-meidated inhibitory effects on these pathways may play a key role in preventing the malignant transformation of ovarian cells in the presence of local serotonin in the ovary. Chronic stress-promoted growth of OC xenografts at the primary sites, where there is relatively high level of HTR1E (Figures 1E-1I), can be inhibited by the injection of extrinsic serotonin, more strongly by specific HTR1E agonist (Figures 7B-7C), supporting that HTR1E mediates a major tumor-suppressive role downstream of serotonin against other serotonin receptor subtypes to protect ovarian cells. However, either the extrinsic serotonin or the specific HTR1E agonist is not able to reduce the stress-promoted formation of peritoneal metastatic nodules and malignant ascites to the level comparable to that in mice without stress stimulation (Figures 7B-7D), possibly due to the dramatically reduced HTR1E expression (Figures 1E-1I, Figures 1F-1G**).** These results suggest that the blockage of HTR1E-mediated tumor-suppressive signaling is a prerequisite for OC metastasis. And reactivation of HTR1E-mediated signaling by its specific agonist may achieve better tumor-suppressive effects than serotonin given the existence of other tumor-promoting serotonin receptor subtypes. For metastasized OC cells, due to the low HTR1E levels, targeting its major downstream signaling mediator SRC could be a promising strategy to for the therapy of OC at advanced stages. Taken together, our results suggest the combination usage of HTR1E specific agonist and SRC inhibitors to be an efficient way to inhibit the growth of OC primary tumor and its peritoneal dissemination.

## Conclusion

Among GPCRs of serotonin in the ovary, highly expressed HTR1E negatively coupled to oncogenic SRC signaling that inhibits SRC-activated downstream pathways including MEK-ERK, PI3K-AKT, and FAK, eventually resulting in restrained cell proliferation, migration and EMT process that favor OC growth and dissemination. Chronic stress-induced decrease of local serotonin in the ovary or decreased HTR1E expression will attenuate HTR1E-mediated inhibitory effect on SRC activation, leading to increased OC growth and dissemination. And reactivation of serotonin/HTR1E signaling by the specific HTR1E agonist or the specific SRC inhibitor is a promising strategy to inhibit chronic stress-promoted OC growth and dissemination.

## Supplementary Material

Supplementary figures and tables.

## Abbreviations

OC: ovarian cancer
5-HT: 5-hydroxytryptamine, serotonin
SSRIs: selective serotonin reuptake inhibitors
MAGeCK: model-based Analysis of Genome-wide CRISPR/Cas9 Knockout
NSG: NOD/SCID
GPCRs: G-protein-coupled receptors
AC: adenylyl cyclase
EMT: epithelial to mesenchymal transition
GSEA: gene set enrichment assay
CUMS: chronic unpredictable mild stress
SRCi: SRC inhibitor
MEKi: MEK inhibitor

## References

[1] Siegel RL, Miller KD, Jemal A (2019). Cancer statistics, 2019. CA: A Cancer Journal for Clinicians.

[2] Watts S, Prescott P, Mason J, McLeod N, Lewith G (2015). Depression and anxiety in ovarian cancer: a systematic review and meta-analysis of prevalence rates. BMJ Open.

[3] Reiche EM, Nunes SO, Morimoto HK (2004). Stress, depression, the immune system, and cancer. Lancet Oncol.

[4] Meraner V, Gamper EM, Grahmann A, Giesinger JM, Wiesbauer P, Sztankay M (2012). Monitoring physical and psychosocial symptom trajectories in ovarian cancer patients receiving chemotherapy. BMC Cancer.

[5] Guereschi MG, Araujo LP, Maricato JT, Takenaka MC, Nascimento VM, Vivanco BC (2013). Beta2-adrenergic receptor signaling in CD4+ Foxp3+ regulatory T cells enhances their suppressive function in a PKA-dependent manner. Eur J Immunol.

[6] Mundy-Bosse BL, Thornton LM, Yang HC, Andersen BL, Carson WE (2011). Psychological stress is associated with altered levels of myeloid-derived suppressor cells in breast cancer patients. Cell Immunol.

[7] Kitajima T, Ariizumi K, Bergstresser PR, Takashima A (1996). A novel mechanism of glucocorticoid-induced immune suppression: the inhibiton of T cell-mediated terminal maturation of a murine dendritic cell line. J Clin Invest.

[8] Yang H, Xia L, Chen J, Zhang S, Martin V, Li Q (2019). Stress-glucocorticoid-TSC22D3 axis compromises therapy-induced antitumor immunity. Nat Med.

[9] Sloan EK, Priceman SJ, Cox BF, Yu S, Pimentel MA, Tangkanangnukul V (2010). The sympathetic nervous system induces a metastatic switch in primary breast cancer. Cancer Res.

[10] Repasky EA, Eng J, Hylander BL (2015). Stress, metabolism and cancer: integrated pathways contributing to immune suppression. Cancer J.

[11] Obradovic MMS, Hamelin B, Manevski N, Couto JP, Sethi A, Coissieux MM (2019). Glucocorticoids promote breast cancer metastasis. Nature.

[12] Dube F, Amireault P (2007). Local serotonergic signaling in mammalian follicles, oocytes and early embryos. Life Sci.

[13] Morch LS, Dehlendorff C, Baandrup L, Friis S, Kjaer SK (2017). Use of antidepressants and risk of epithelial ovarian cancer. Int J Cancer.

[14] Torre LA, Trabert B, DeSantis CE, Miller KD, Samimi G, Runowicz CD (2018). Ovarian cancer statistics, 2018. CA Cancer J Clin.

[15] Dizeyi N, Bjartell A, Nilsson E, Hansson J, Gadaleanu V, Cross N (2004). Expression of serotonin receptors and role of serotonin in human prostate cancer tissue and cell lines. Prostate.

[16] Sarrouilhe D, Clarhaut J, Defamie N, Mesnil M (2015). Serotonin and cancer: what is the link?. Curr Mol Med.

[17] Forbes NF, Stewart CA, Matthews K, Reid IC (1996). Chronic mild stress and sucrose consumption: validity as a model of depression. Physiol Behav.

[18] Shalem O, Sanjana NE, Hartenian E, Shi X, Scott DA, Mikkelson T (2014). Genome-scale CRISPR-Cas9 knockout screening in human cells. Science.

[19] Lengyel E, Burdette JE, Kenny HA, Matei D, Pilrose J, Haluska P (2014). Epithelial ovarian cancer experimental models. Oncogene.

[20] Song M, Yeku OO, Rafiq S, Purdon T, Dong X, Zhu L (2020). Tumor derived UBR5 promotes ovarian cancer growth and metastasis through inducing immunosuppressive macrophages. Nat Commun.

[21] Li W, Xu H, Xiao T, Cong L, Love MI, Zhang F (2014). MAGeCK enables robust identification of essential genes from genome-scale CRISPR/Cas9 knockout screens. Genome Biol.

[22] Sinha D, Chong L, George J, Schluter H, Monchgesang S, Mills S (2016). Pericytes Promote Malignant Ovarian Cancer Progression in Mice and Predict Poor Prognosis in Serous Ovarian Cancer Patients. Clin Cancer Res.

[23] Mitra AK, Davis DA, Tomar S, Roy L, Gurler H, Xie J (2015). *In vivo* tumor growth of high-grade serous ovarian cancer cell lines. Gynecol Oncol.

[24] Taki M, Abiko K, Baba T, Hamanishi J, Yamaguchi K, Murakami R (2018). Snail promotes ovarian cancer progression by recruiting myeloid-derived suppressor cells via CXCR2 ligand upregulation. Nat Commun.

[25] Tothill RW, Tinker AV, George J, Brown R, Fox SB, Lade S (2008). Novel molecular subtypes of serous and endometrioid ovarian cancer linked to clinical outcome. Clin Cancer Res.

[26] Cancer Genome Atlas Research N (2011). Integrated genomic analyses of ovarian carcinoma. Nature.

[27] Ma Y-C, Huang X-Y (1998). Identification of the binding site for Gqα on its effector Bruton's tyrosine kinase. Proceedings of the National Academy of Sciences.

[28] Ma Y-C, Huang J, Ali S, Lowry W, Huang X-Y (2000). Src Tyrosine Kinase Is a Novel Direct Effector of G Proteins. Cell.

[29] Nordquist N, Oreland L (2010). Serotonin, genetic variability, behaviour, and psychiatric disorders-a review. Ups J Med Sci.

[30] Healy D (2015). Serotonin and depression. BMJ: British Medical Journal.

[31] Clausell DE, Soliman KF (1978). Ovarian serotonin content in relation to ovulation. Experientia.

[32] Nikishin DA, Alyoshina NM, Semenova ML, Shmukler YB (2019). Analysis of Expression and Functional Activity of Aromatic L-Amino Acid Decarboxylase (DDC) and Serotonin Transporter (SERT) as Potential Sources of Serotonin in Mouse Ovary. Int J Mol Sci.

[33] Min L, Jiajie H, Tian M, Shi W, Hong D (2011). Application of a disposable screen-printed electrode to depression diagnosis for laboratory rats based on blood serotonin detection. Analytical sciences: the international journal of the Japan Society for Analytical Chemistry.

[34] Xia L, Yuan F, Shifu X, Sufang P, Xiaowei D, Xianjie Z (2015). Decreased platelet 5-hydroxytryptamin (5-HT) levels: a response to antidepressants. Journal of affective disorders.

[35] Katz RJ (1982). Animal model of depression: pharmacological sensitivity of a hedonic deficit. Pharmacol Biochem Behav.

[36] Hill MN, Hellemans KG, Verma P, Gorzalka BB, Weinberg J (2012). Neurobiology of chronic mild stress: parallels to major depression. Neurosci Biobehav Rev.

[37] Willner P (2017). The chronic mild stress (CMS) model of depression: History, evaluation and usage. Neurobiol Stress.

[38] Davis M (1992). The role of the amygdala in fear and anxiety. Annu Rev Neurosci.

[39] de Kloet ER, Joels M, Holsboer F (2005). Stress and the brain: from adaptation to disease. Nat Rev Neurosci.

[40] McKune CM, Watts SW (2001). Characterization of the serotonin receptor mediating contraction in the mouse thoracic aorta and signal pathway coupling. J Pharmacol Exp Ther.

[41] Janssen P, Tack J, Sifrim D, Meulemans AL, Lefebvre RA (2004). Influence of 5-HT1 receptor agonists on feline stomach relaxation. Eur J Pharmacol.

[42] Zuo X, Chen Z, Cai J, Gao W, Zhang Y, Han G (2019). 5-Hydroxytryptamine Receptor 1D Aggravates Hepatocellular Carcinoma Progression Through FoxO6 in AKT-Dependent and Independent Manners. Hepatology.

[43] Gautam J, Banskota S, Regmi SC, Ahn S, Jeon YH, Jeong H (2016). Tryptophan hydroxylase 1 and 5-HT7 receptor preferentially expressed in triple-negative breast cancer promote cancer progression through autocrine serotonin signaling. Mol Cancer.

[44] Masson J, Emerit MB, Hamon M, Darmon M (2012). Serotonergic signaling: multiple effectors and pleiotropic effects. Wiley Interdisciplinary Reviews: Membrane Transport and Signaling.

