# Supplementary material for: Serotonin/HTR1E signaling blocks chronic stress-promoted progression of ovarian cancer
Source: Theranostics. 2021 May 8;11(14):6950–65. doi: 10.7150/thno.58956 (PMC8171092; doi:10.7150/thno.58956)
Supplement: Supplementary file 1 — Supplementary figures and tables. [file thnov11p6950s1.pdf]

Table S1 Primers used in this study.

Primer	Forward Sequence （5’→3’）	Reverse Sequence （5’→3’）
Human		
HTR1A	GACGTGACCGTCAGCTACCA	TCGAGGGCGATGAACAGGTC
HTR1B	CTGCTGGTTCCACCTAGCCA	TTGGTCCCCAAAGGTCGCTT
HTR1D	CTCCAACAGATCCCTGAATGC	CCTGGTGAGTAAGATGGTGGT
HTR1E	CTCCCAGGTTCTGTCTCGCC	TGAAGGTTCCTTGTGGACGGT
HTR1F	CTGGAGGGCGTTTCAAGAGC	TGCAGCGATCACAAGGGAGT
HTR2A	CAGGATCCTAGCAGTGCGGG	TTCTCACCAAACCGAGGACAAA
HTR2B	GAGGGGGAACTCTCTGGCAT	CCATGCCAAACACTCAAAAGCC
HTR2C	TCTTCAGTGCCAAAGGGTGGA	TGGCCAGTTTTGTACCCCGT
HTR6	GGGAACTCTTGGTCGCCAGT	CAGACCTGGGGGATAGGGGA
HTR7	GAGCTGCTGCTGCGAACTG	GGCTTCACCTCACCGGTTCC
β-actin	CGTCACCAACTGGGACGA	ATGGGGGAGGGCATACC
Mouse		
TPH1	GAGTCCCGGAAATCAAAGCAAAG	GGTGGTCGGCGTCAAGTTCG
TPH2	TTGCCGGGAGTACCTGAAAAACC	TCCGAAACAAAGTAAGCGTCCTG
DDC	CCCCCAGGAGCCAGAAACATAC	CCTGCAGCTGGCGGATAACTT
SERT	AGTACAAGCGCTGGGGATGAAG	GGAGGCGATATAAAAGGCAATGA
VMAT1	AGATGGGTCGGTGGCTGTGCTC	CTTCGTGGGCCTCTGGATTGTGTA
VMAT2	TGCCCCAGTGAAGACAAAGACCT	CATGCCCATCCCAGCCACAG
β-actin	GGCTGTATTCCCCTCCATCG	GCACAGGGTGCTCCTCAG

Table S2 Antibodies used in this study.

Antigens	Manufacturers
HTR1E(Anti-S31)	ab236651, Abcam, Cambridge,
SRC	11097-1-AP, Proteintech,
p-Src (Y418)	ab4816, Abcam, Cambridge, MA,
AKT	10176-2-AP, Proteintech,
p-AKT (Ser473)	#4060, Cell Signaling Technology,
FAK	12636-1-AP, Proteintech,
p-FAK（Tyr397）	#8556, Cell Signaling Technology,
Serotonin	S5545, Sigma-Aldrich, Shanghai,
ERK1/2	#4695, Cell Signaling Technology,
ERK1/2 (Thr 202/Tyr 204)	bs-3016R, Bioss, Beijing, China
Vimentin	ab92547, Abcam, Cambridge,
Vimentin	ab8978, Abcam, Cambridge, MA,
E-cadherin	#14472, Cell Signaling
β-actin	#3700, Cell Signaling Technology,
Goat-anti-Rabbit-HRP antibody	Zhongshanjinqiao, Beijing
Goat-anti-Mouse-HRP antibody	Zhongshanjinqiao, Beijing

Table S3 Top ten genes screened by Genome-wide CRISPR/Cas9 Knckout (MAGeCK) algorithm.

Rank	Gene	P-value	Number of sgRNA	$\log_2$(Peri/Pri)
1	hsa-mir-6738	0.00041299	2	11.342
2	ADAMTS4	0.00085422	2	11.605
3	ZBTB26	0.001384	2	10.212
4	RPS6KA6	0.0020191	2	9.9735
5	RWDD3	0.0025291	2	8.7734
6	TMEM56	0.0025291	2	8.7734
7	hsa-mir-517b	0.0034329	2	7.004

Rank	Gene	P-value	Number of sgRNA	log_2(Peri/Pri)
8	TEX14	0.0038772	3	4.4379
9	hsa-mir-6886	0.0042451	2	6.2488
10	HTR1E	0.0061872	2	6.0176

Pri, primary OC xenografts

Peri, peritoneal disseminated nodules

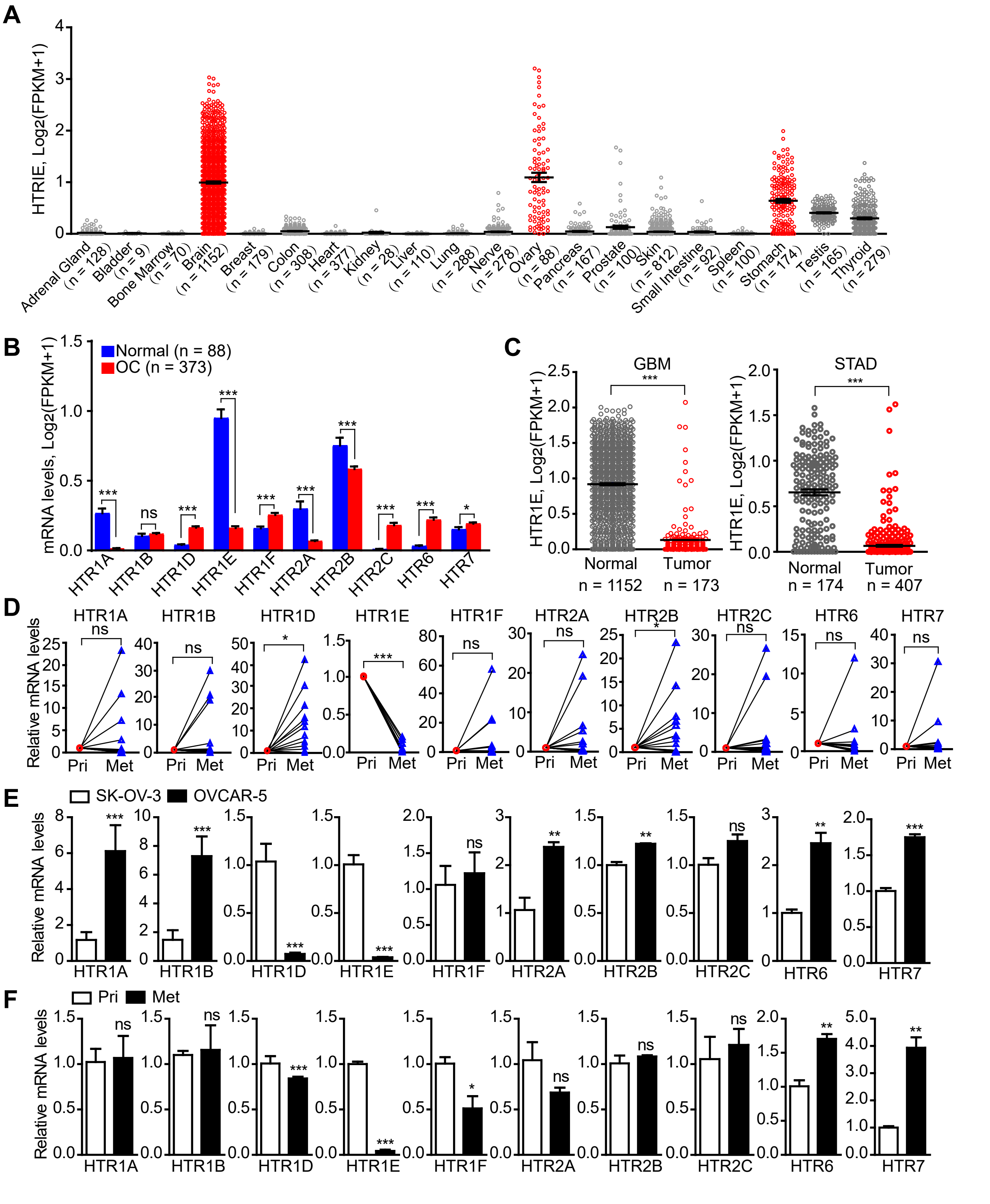

Figure S1. HTR1E is specifically expressed in normal human ovary and is decreased in OC. (A) RNA sequencing analysis of HTR1E expression in 20 different tissues from GTEx database. **(B)** RNA sequencing analysis of the expression of indicated serotonin receptors in normal human ovary (n = 88) and OC tissues (n = 373). Data are from TCGA and GTEx databases. **(C)** RNA sequencing analysis of HTR1E expression in human Glioblastoma (GBM) and stomach adenocarcinoma (STAD). Data are from TCGA GBM/STAD patients database and GTEx normal human brain/stomach tissue database. **(D)** qRT-PCR analysis of indicated serotonin receptors in human primary OC samples and paired peritoneal metastases (n = 13, *P < 0.05, ns not significant, by paired, two-tailed student's t-test). **(E)** qRT-PCR analysis of the serotonin receptors in SK-OV-3 and OVCAR-5 cells. Data are shown as means ± SEM from three independent experiments; **P < 0.01, ***P < 0.001, ns not significant, by unpaired, two-tailed student's t-test. **(F)** qRT-PCR analysis of the serotonin receptors in the primary OC xenografts and the peritoneal metastases dissected from SK-OV-3 orthotopic murine model of OC. Data are shown as means ± SEM, *P < 0.05, **P < 0.01, ***P < 0.001, ns not significant, by unpaired, two-tailed student's t-test.

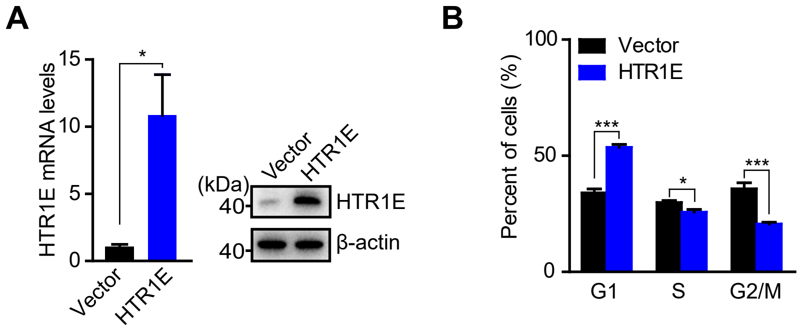

Figure S2. The effects of serotonin on cell-cycle in OVCAR-5 cells with HTR1E overexpression. (A) qRT-PCR and western blot analysis to confirm the reconstituted expression of HTR1E in OVCAR-5 cells (Data are shown as means ± SEM from three independent experiments; $^*P < 0.05$, by unpaired, two-tailed student's t-test). **(B)** Overexpression of HTR1E induced cell-cycle arrest at the S and G2/M phase in OVCAR-5 cells (means ± SEM from three independent experiments; $^*P < 0.05$, $^{***}P < 0.001$, by unpaired, two-tailed student's t-test).

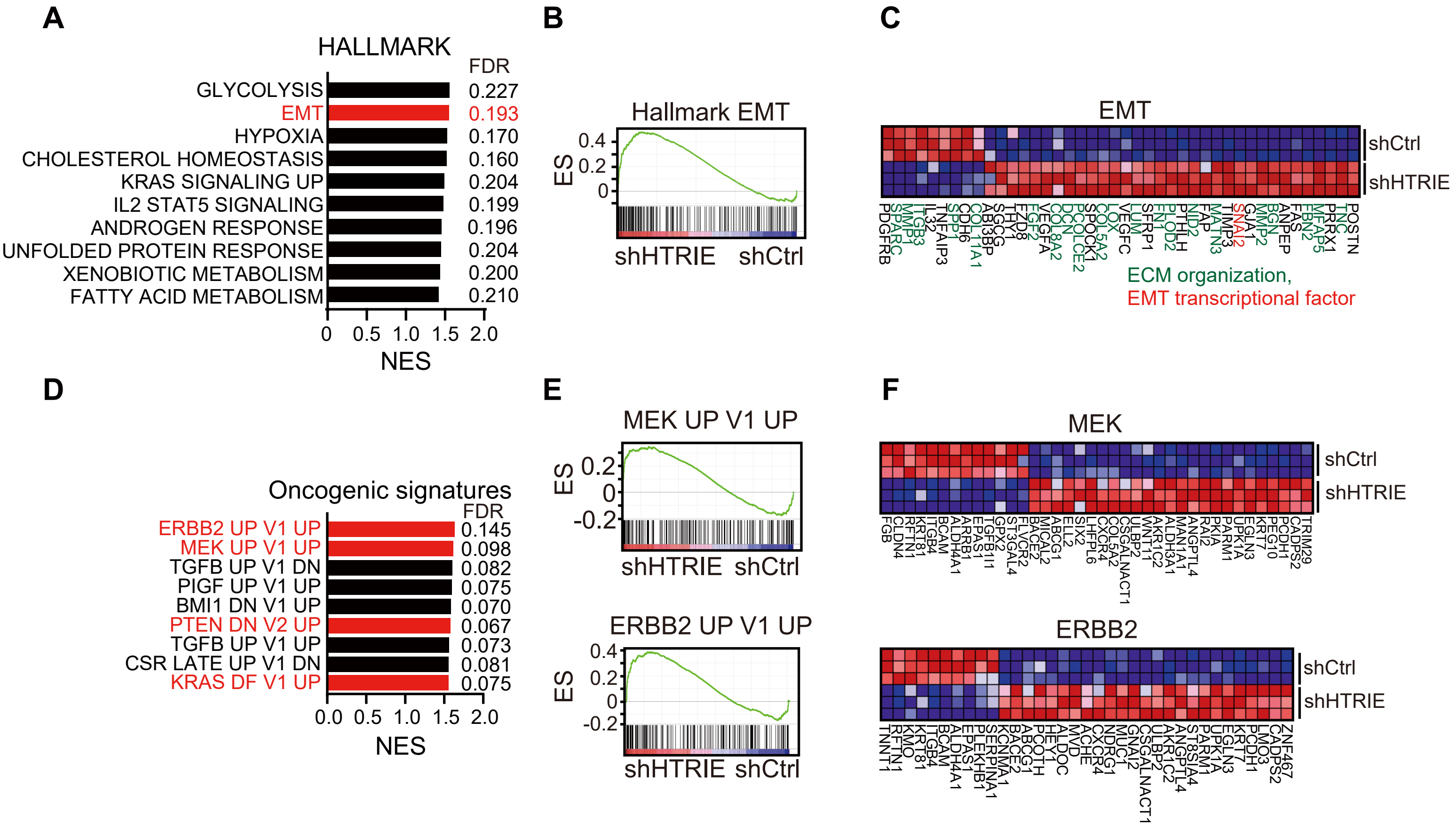

Figure S3. RNA sequencing identifies genes regulated by HTR1E. (A-C) Top ten hallmark pathways in which the upregulated genes in shHTR1E-transfected SK-OV-3 cells are enriched when analyzed by gene set enrichment analysis (GSEA) **(A)**, and the GSEA results of epithelial mesenchymal transition (EMT) hallmark pathway **(B)** and the heatmap of related genes with over 2-fold change upon knocking down HTR1E **(C)**. **(D-F)** Top oncogenic signatures that are most likely triggered by shHTR1E treatment in SK-OV-3 cells shown by GSEA **(D)**, and the GSEA enrichment of HTR1E-regulated genes in oncogenic MEK- and ERBB2- signature genes **(E)** and the heatmap of relevant genes with over 2-fold change in shHTR1E treated cells **(F)**.

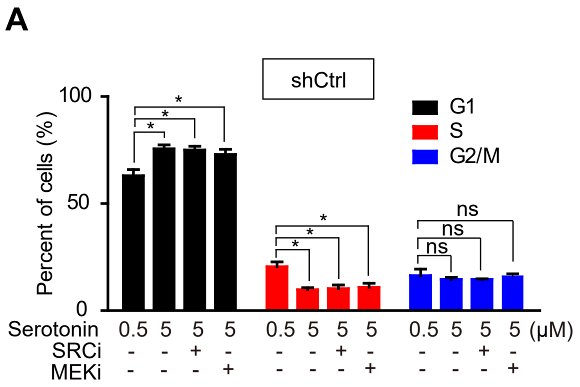

Figure S4. The effects of serotonin, SRCi and MEKi on cell-cycle in shHTR1E or shCtrl SK-OV-3 cells. (A-B) Flow cytometric analyses were conducted to examine the cell-cycle of control SK-OV-3 cells **(A)** and HTR1E-silenced SK-OV-3 cells **(B)** in the presence of indicated dosages of serotonin, SRCi and MEKi. The quantification plots are shown as means ± SEM from three independent experiments. (*P < 0.05, ***P < 0.001, ns not significant, by unpaired, two-tailed student's t-test).

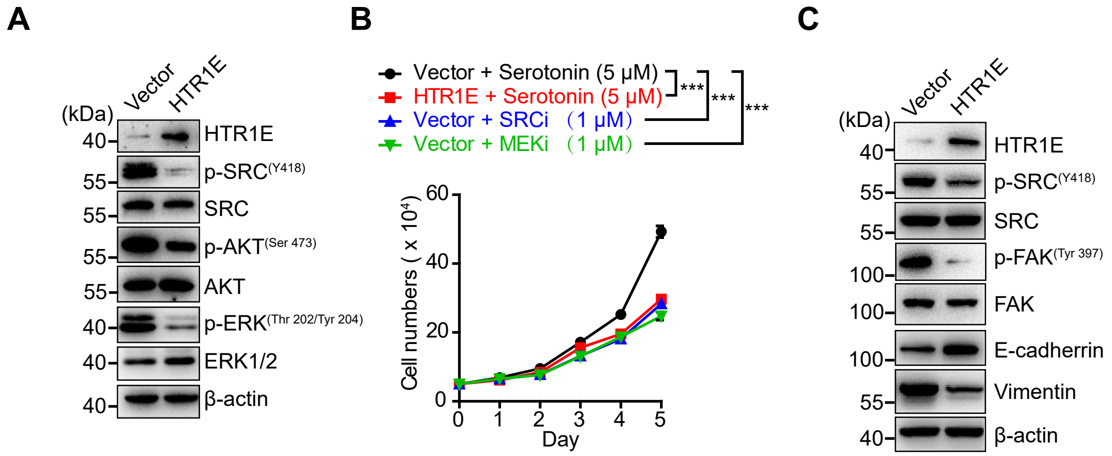

Figure S5. Reconstitution of HTR1E in OVCAR-5 cells inhibits SRC-mediated activation of ERK and EMT. (A) Western blot analysis of HTR1E-reconsitituted and control OVCAR-5 cells for the activation of SRC, AKT and ERK in the presence of 5 μM serotonin. **(B)** Cell proliferation curves of HTR1E-reconstituted OVCAR-5 cells and control cells in the presence of serotonin (5 μM) and SRCi (1 μM) or MEKi (1 μM). Data are shown as means ± SEM from three independent experiments; ***$P < 0.001$, by two-way ANOVA test. **(C)** Western blot analysis of EMT markers in HTR1E-reconstituted OVCAR-5 cells in the presence of 5 μM serotonin.

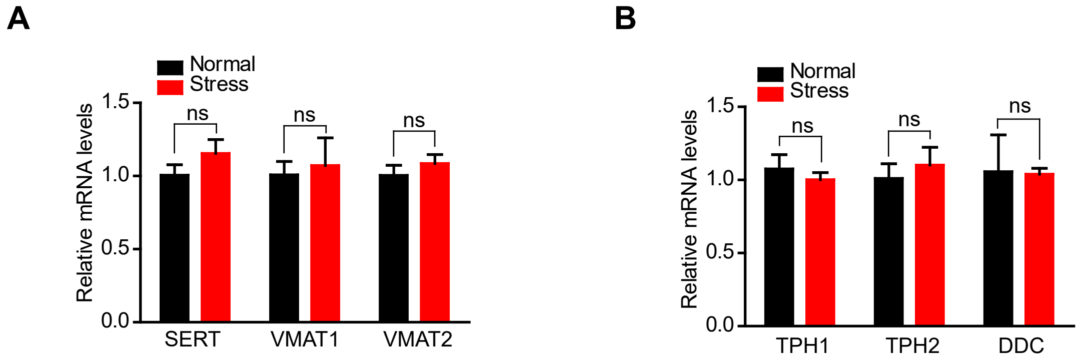

Figure S6. Gene expression of serotonin transportation and enzymes of serotonin synthesis in mice ovary. **(A)** qRT-PCR analysis of serotonin transporter SERT, monoamine vesicular transporters VMAT1 and VMAT2 in mice with or without stress stimulation for 21 days. **(B)** qRT-PCR analysis of enzymes of serotonin synthesis TPH1, TPH2 and DDC in mice with or without stress stimulation for 21 days. (All the quantification results were shown as means ± SEM; ns not significant, by unpaired, two-tailed student's t-test.)

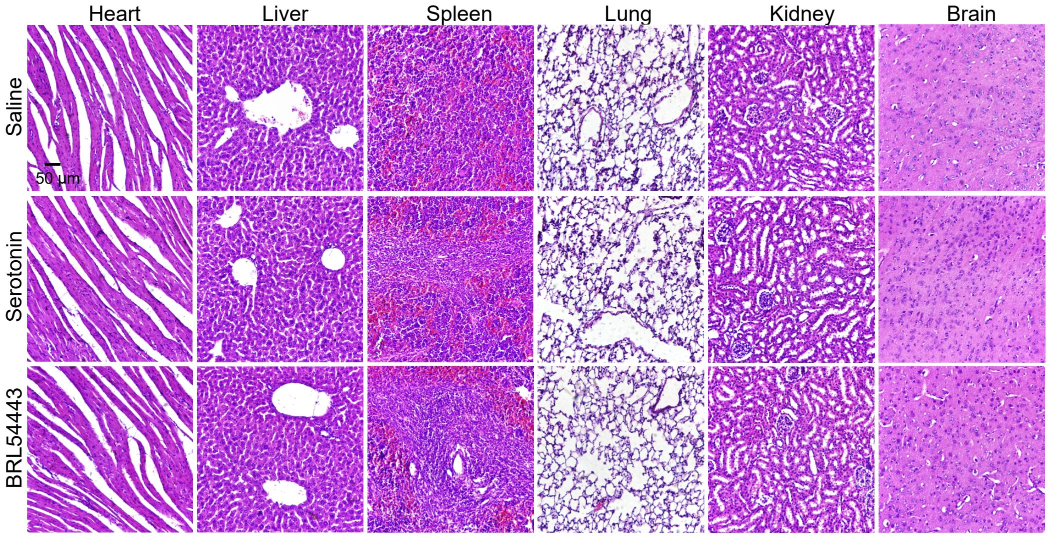

Figure S7. The toxicities of serotonin and HTR1E agonist BRL54443 in mice. H&E staining of major organs of mice treated with saline, serotonin (5 mg/kg), and specific HTR1E agonist BRL54443 (10 mg/kg).
